# Plant but not animal sourced nitrate intake is associated with lower dementia-related mortality in the Australian Diabetes, Obesity, and Lifestyle Study

**DOI:** 10.3389/fnut.2024.1327042

**Published:** 2024-08-21

**Authors:** Anjana Rajendra, Nicola P. Bondonno, Liezhou Zhong, Simone Radavelli-Bagatini, Kevin Murray, Stephanie R. Rainey-Smith, Samantha L. Gardener, Lauren C. Blekkenhorst, Dianna J. Magliano, Jonathan E. Shaw, Robin M. Daly, Kaarin J. Anstey, Joshua R. Lewis, Jonathan M. Hodgson, Catherine P. Bondonno

**Affiliations:** ^1^Nutrition and Health Innovation Research Institute, School of Medical and Health Sciences, Royal Perth Hospital Research Foundation, Edith Cowan University, Perth, WA, Australia; ^2^The Danish Cancer Society Research Centre, Copenhagen, Denmark; ^3^School of Population and Global Health, University of Western Australia, Perth, WA, Australia; ^4^Centre for Healthy Ageing, Health Futures Institute, Murdoch University, Murdoch, WA, Australia; ^5^Centre of Excellence for Alzheimer's Disease Research and Care, School of Medical and Health Sciences, Edith Cowan University, Joondalup, WA, Australia; ^6^Australian Alzheimer's Research Foundation, Perth, WA, Australia; ^7^School of Psychological Science, University of Western Australia, Perth, WA, Australia; ^8^Lifestyle Approaches Towards Cognitive Health Research Group, Murdoch University, Murdoch, WA, Australia; ^9^Medical School, The University of Western Australia, Royal Perth Hospital, Perth, WA, Australia; ^10^Diabetes and Population Health, Baker Heart and Diabetes Institute, Melbourne, VIC, Australia; ^11^Clinical Diabetes and Epidemiology, Baker Heart and Diabetes Institute, Melbourne, VIC, Australia; ^12^School of Public Health and Preventive Medicine, Monash University, Melbourne, VIC, Australia; ^13^Institute for Physical Activity and Nutrition, School of Exercise and Nutrition Sciences, Deakin University, Geelong, VIC, Australia; ^14^School of Psychology, The University of New South Wales, Sydney, NSW, Australia; ^15^Neuroscience Research Australia (NeuRA), Randwick, NSW, Australia; ^16^UNSW Ageing Futures Institute, Kensington, NSW, Australia; ^17^Centre for Kidney Research, Children’s Hospital at Westmead, School of Public Health, Sydney Medical School, The University of Sydney, Sydney, NSW, Australia

**Keywords:** dietary nitrate, dementia, diet, diabetes, cohort

## Abstract

**Introduction:**

Dietary nitrate is potentially beneficial for cardiovascular, cerebrovascular, and nervous systems due to its role as a nitric oxide (NO) precursor. Increased nitrate intake improves cardiovascular health and therefore could protect against dementia, given the cardiovascular-dementia link.

**Objective:**

To investigate the association between source-dependent nitrate intake and dementia-related mortality. As individuals with diabetes are at higher risk of dementia, a secondary aim was to investigate if the associations between nitrate and dementia varied by diabetes mellitus (DM) and pre-diabetes status.

**Methods:**

This study involved 9,149 participants aged ≥25 years from the well-characterised Australian Diabetes, Obesity, and Lifestyle (AusDiab) Study followed over a period of 17 years. Intakes of plant-sourced, vegetable-sourced, naturally occurring animal-sourced nitrate, and processed meat (where nitrate is an allowed additive)-sourced nitrate were assessed from a 74-item food frequency questionnaire completed by participants at baseline and nitrate databases were used to estimate nitrate from these different dietary sources. Associations between source-dependent nitrate intake and dementia-related mortality were assessed using multivariable-adjusted Cox proportional hazards models adjusted for demographics, lifestyle, and dietary factors.

**Results:**

Over 17 years of follow-up, 93 (1.0%) dementia-related deaths occurred of 1,237 (13.5%) total deaths. In multivariable-adjusted models, participants with the highest intakes of plant-sourced nitrate (median intake 98 mg/day) had a 57% lower risk of dementia-related mortality [HR (95% CI): 0.43 (0.22, 0.87)] compared to participants with lowest intakes of plant-sourced nitrate (median intake 35 mg/day). A 66% lower risk was also seen for higher intakes of vegetable-sourced nitrate [HR (95% CI): 0.34 (0.17, 0.66)]. No association was observed for animal-sourced nitrate, but the risk was two times higher amongst those who consumed the most processed meat-sourced nitrate intake [HR (95%): 2.10 (1.07, 4.12)]. The highest intake of vegetable-sourced nitrate was associated with a lower risk of dementia-related mortality for those with and without DM and pre-diabetes.

**Conclusion:**

Encouraging the intake of nitrate-rich vegetables, such as green leafy vegetables and beetroot, may lower the risk of dementia-related mortality, particularly in individuals with (pre-) diabetes who are at a higher dementia risk.

## 1 Introduction

Dementia is a leading cause of mortality globally (1) and is the second leading cause of death in Australia (2). Currently, more than 55 million people have dementia with this number predicted to surge to 152 million by 2050 (3). With no cure for dementia discovered to date, identifying evidence-based preventive strategies to reduce the risk of dementia is a global research priority. Targeting modifiable risk factors is a strategy that is estimated, using population-attributable risk models, to prevent or delay up to 40% of dementia cases (4). Of the 12 modifiable risk factors identified by Lancet Commission, 5 [high blood pressure, obesity, alcohol intake, diabetes mellitus (DM), and depression] can be positively impacted by a healthy diet (4). Moreover, WHO guidelines include diet as a modifiable risk factor as do systematic reviews (5). Thus, identifying and promoting higher intakes of the protective components of a healthy dietary pattern could prevent or delay the onset of dementia.

One such potential protective dietary component is nitrate. Nitrate has been identified as an important exogenous source of the cellular signalling molecule, nitric oxide (NO) (6). NO plays a fundamental role in the regulation of the cardiovascular (7), cerebrovascular (8), and the central nervous systems (9). Increasing NO through dietary nitrate intake positively impacts the cardiovascular system (10), and could also potentially impact the cerebrovascular and central nervous systems.

We have recently shown that habitual intake of dietary nitrate from sources where nitrate is naturally present impacts cognitive performance, amongst cognitively unimpaired older adults in an apolipoprotein E *(APOE)* genotype contingent manner. Specifically, higher intake of dietary nitrate was associated with better language scores in non-carriers of the *APOE* ε4 allele and with better episodic recall and recognition memory in those at higher risk of Alzheimer’s disease (AD; the most common form of dementia) due to the presence of one or two *APOE* ε4 alleles (11). Given the role of NO in the cerebrovascular, and central nervous systems, the established benefit of dietary nitrate on cardiovascular health, and the recognised vascular contributions to dementia, there is a strong rationale to investigate whether habitual intake of dietary nitrate may also impact risk of dementia-related mortality.

Nitrate is found in high concentrations in green leafy vegetables and some root vegetables, contributing ~70–80% to total dietary nitrate intake (12). Nitrate is also found naturally in meat and other animal products, contributing ~10–15% to total dietary nitrate intake (12). In contrast nitrite concentration is very low in plants and higher in meat (13). Furthermore, nitrate and nitrite are both highly regulated preservatives used in processed meat, contributing to ~5% total dietary nitrate intake (14). Nitrate from plant sources has been shown to increase NO with beneficial health effects (14). In contrast, nitrate, through conversion to nitrite, also has the potential to form carcinogenic (15) and neurodegenerative *N*-nitrosamines (16) exogenously and endogenously. Dietary nitrate’s potential favourable (increases NO) and adverse (forms *N-*nitrosamines) effects are hypothesised to be source dependent (14). Plant-sourced nitrate intake is accompanied by other beneficial components such as vitamin C, flavonoids, and antioxidants that can inhibit nitrosamine production (17). The presence of nitrate and nitrite in processed meat is hypothesised to contribute to the negative health effects of processed meat intake.

The primary aim of this study was to investigate the association between habitual intakes of nitrate from different sources and dementia-related mortality in the Australian Diabetes, Obesity, and Lifestyle (AusDiab) Study. The sources of nitrate were (i) plant-sourced nitrate, (ii) vegetable-sourced nitrate, (iii) animal-sourced nitrate (excluding nitrate additives), and (iv) processed meat-sourced nitrate (including nitrate additives, but excluding fresh sausages, i.e., meat where nitrate is an allowed additive). As individuals with diabetes have a higher risk of developing dementia (18, 19), a secondary aim was to explore whether dietary nitrate was associated with fewer dementia-related mortality in participants with DM and pre-diabetes (with either impaired glucose tolerance or impaired fasting glucose).

## 2 Methods

### 2.1 Study population

The Australian Diabetes, Obesity, and Lifestyle (AusDiab) Study is a population-based longitudinal study of adults (aged ≥25 years), which recruited 11,247 men and women across Australia in 1999–2000. Further details regarding methods and response rates are described previously (20). The AusDiab study was approved by the International Diabetes Institute ethics committee and informed consent was obtained from all the participants (20).

Participants were excluded from the current study if they had implausible energy intakes [*n* = 546 (<2,500 kJ/day or > 14,500 kJ/day for females and < 3,300 kJ/day or 17,500 kJ/day for males)] (21, 22), whilst participants who were pregnant were not excluded from AusDiab during recruitment, they were excluded from the current study if they were pregnant at the time of recruitment (*n* = 60) as diet may be changed during pregnancy, had chronic renal disease (estimated glomerular filtration rate < 60; *n* = 899) (23, 24), or if they had missing or implausible values for covariates (*n* = 593). Thus, this study included 9,149 participants in the analyses.

### 2.2 Exposures

At baseline and 5 years, participants reported their usual intakes of 74 food and beverage items over the previous 12 months via a 74-item Cancer Council of Victoria Food Frequency Questionnaire (CCVFFQ) (25, 26). From these items, habitual intakes of different sources of dietary nitrate where nitrate is naturally present (plant-sourced nitrate, vegetable-sourced nitrate, and animal-sourced nitrate) and where nitrate is used as an additive for most products except fresh sausage (processed meat) (27), were assessed and quantified as described below. Baseline FFQ data were used for analyses as 5-year FFQ data was only available for 60% of the cohort.

#### 2.2.1 Plant- and vegetable-sourced nitrate intake

A comprehensive plant-based food reference nitrate database with nitrate values from 304 plant-based foods from 64 countries was used to calculate nitrate values of all plant-based foods; vegetables, fruits, cereals, herbs, spices, pulses, and nuts (28). The nitrate content of plant foods differs depending on the country of cultivation; therefore, the following strategy was employed (12): the median value for each plant food was used if there were three or more references in the database for Australia; the median of values for all Oceania (Australia, New Zealand, and surrounding islands) was used if there were fewer than three references the database for Australia; the median of values for all countries in the database was used if there were fewer than three references available for Oceania. The median nitrate value (mg/g) of each plant-based food was multiplied by the estimated quantity of the plant-based food consumed (g/day). To take into account the effect of cooking, for cooked plant-based foods the assigned nitrate value was reduced by 50% (28). The nitrate values of each individual plant-based and vegetable-based food were summed to obtain total plant-sourced nitrate and total vegetable-sourced nitrate consumed per day.

#### 2.2.2 Animal-sourced nitrate intake

Red meat, dairy, seafood, eggs, and poultry were used for the calculation of naturally occurring animal-sourced nitrate intake. Processed meat, where nitrate is as an allowed additive for most products except fresh sausage, was calculated separately due its link with detrimental health effects (29). To calculate animal-sourced nitrate intake, a recently published nitrate reference database for animal-sourced food products, with data from 51 countries, was used (13). The same strategy as described above for plant-based foods and vegetables was employed. However, the 50% reduction in value was not applied to animal products because the majority of the data sources in the animal database did not indicate cooking method clearly. To determine total animal-sourced nitrate consumed (mg/day), the amount of the specific animal-sourced food consumed (g/day) was multiplied by its median nitrate content (mg/g).

#### 2.2.3 Total nitrate intake

The sum of nitrate intake from all food items in the FFQ including discretionary foods such as chocolate, biscuits, pizza, and crisps were used to compute total nitrate intake (mg/day). The amount of food item consumed (g/day) was multiplied by the assigned median nitrate value (mg/g) for that food item to calculate nitrate intake (mg/day). If the nitrate value for that food item was not available in any of the above listed databases, the value of zero was assigned.

### 2.3 Study outcomes

Dementia-related mortality, defined as death with mention of a dementia diagnosis on any part of the death certificate, was the primary outcome of this study. Information on dates and causes of death were obtained from National Death Index (NDI), using the *International Classification of Diseases, Tenth Revision* (ICD-10) Australian Modification format (30). Death due to dementia was defined using the ICD-10 codes; F00 (Alzheimer’s disease), G30 (Alzheimer’s disease), F01 (vascular dementia) and F03 (unspecified dementia), regardless of whether they were underlying or secondary causes of death.

### 2.4 Covariates

Demographic data including age, sex (male/female), education level (never to some high school, completed University, or equivalent), marital status (never married, married, *de-facto*, separated, divorced, and widowed), smoking status, alcohol intake, physical activity, and weekly income ($0–199, $200–399, $400–599, $600–799, $800–1,499, and $1500+) were collected at local testing centres by questionnaire at baseline. Smoking status was classified as: never smoked (<100 cigarettes in lifetime), ex-smoker (not daily for at least the previous 3 months), and current smoker (smoking daily) (31). The Active Australia Survey Questionnaire was used to record self-reported physical activity routine in the past week as described previously (32, 33). Physical activity levels were categorised as sedentary (zero physical activity), insufficient (<150 min/week), and sufficient (>150 min/week). All the participants undertook a 75-g oral glucose tolerance test except those who were pregnant or taking prescribed hypoglycaemic medication. An Olympus AU600 analyser (Olympus Optical, Tokyo, Japan) was used to measure fasting plasma glucose (FPG), 2-h plasma glucose (2-h PG), and fasting serum total cholesterol. Participants were categorised as having known diabetes mellitus (KDM) if they reported of having prescribed hypoglycaemic medication by a physician or had FPG ≥ 7.0 mmol/L or 2-h PG ≥ 11.1 mmol/L. Participants who did not report of having DM but had a FPG ≥ 7.0 mmol/L or 2-h PG ≥ 11.1 mmol/L were categorised as having newly diagnosed diabetes mellitus (NDM). Amongst those diagnosed as KDM, 92% had type 2 DM Participants were categorised as having: (1) impaired fasting glucose (IFG) if their FPG was ≥6.1 mmol/L and < 7. mmol/L and their 2-h PG was <7.8 mmol/L; (2) impaired glucose tolerance (IGT) if their 2-h PG was ≥7.8 mmol/L and < 11.1 mmol/L and their FPG < 7.0 mmol/L; and (3) normal glucose tolerance if their FPG was <6.1 mmol/L and their 2-h PG was <7.8 mmol/L. Participants with either IFG or IGT were classified as having pre-diabetes (20, 34). Height was measured without shoes using a stadiometer and was rounded to the nearest 0.5 cm. A mechanical beam balance was used to measure weight without shoes and extra clothing (35). Body mass index (BMI) was calculated by dividing weight (kg) by height (squared metres). Anthropometric details have been described previously (20, 36). Relative socio-economic situations of geographic area were used to calculate Socio-Economic Indices For Areas (SEIFA) based on 5 yearly censuses from 1999 (37). Intakes of dietary covariates were captured from the FFQ as stated previously.

### 2.5 Statistical analysis

Statistical analyses were performed using Stata version 15 (StataCorp, College Station, Texas 77845, United States). For all tests, statistical significance was set at *p* ≤ 0.05 (two-tailed). Participants were followed up for a period of 17 years from the date of study enrolment until death, or until end of follow-up date, 17 April 2017, whichever came first. Cox proportional hazards models were used to estimate the association between baseline habitual intake of plant-sourced, vegetable-sourced, naturally occurring animal-sourced, and processed meat-sourced nitrate and dementia-related mortality up to 17 years of follow-up. We investigated whether associations were non-linear using restricted cubic splines but *p*-values from likelihood ratio tests comparing appropriate nested models were all *p* > 0.05. Thus, hazard ratios (HRs) and 95% confidence intervals were attained from the models with exposure fitted as quartiles. The proportional hazards assumption was tested based on Schoenfeld residuals with no global violation of the assumption found. Covariates were selected *a priori based* on the current knowledge of confounding factors of nitrate intake and dementia. We used four models of adjustment: Model 1 included age and sex; Model 2 included age, sex, BMI, physical activity, smoking status (never/former/current), education level, marital status, income, SEIFA, alcohol consumption, serum cholesterol levels, presence of DM, and/or pre-diabetes; Model 3a included all the covariates adjusted for Model 2 plus energy intake; and when plant- and vegetable-sourced nitrate were the exposures of interest, Model 3b included all the covariates adjusted for in Model 2 plus potential dietary confounding variables such as intakes (g/day) of red meat, fish, saturated fatty acids, polysaturated fatty acids, monosaturated fatty acids and when naturally occurring animal and processed meat-sourced nitrate were the exposures of interest, Model 3b included all covariates in Model 2, plus intake of saturated fatty acids, polysaturated fatty acids, monosaturated fatty acids, and vegetables. We performed sensitivity analyses to check the robustness of the association of plant and vegetable-sourced nitrate with dementia-related mortality by adjusting for other factors such as intakes of flavonoids, vitamin C, and fibre. Additionally, we carried out stratified analyses by pre-diabetes and DM status at baseline to explore possible effect modification. We also investigated interactions with established risk factors for dementia, namely DM and sex. Logistic regression models were performed to obtain 17-year predicted absolute risk estimates for dementia in individuals with/without DM and pre-diabetes. An exploratory analysis was run to determine if there were any substantial changes in the intakes of dietary nitrate from different dietary sources in the participants with diet data at baseline and 5-year follow-up.

## 3 Results

### 3.1 Baseline characteristics

The study cohort (*n* = 9,149) was comprised of 46.7% males, had a median [IQR] age of 49 [40–59] years at study enrolment, and had a median [IQR] follow-up time of 16 [16–17] years. The median [IQR] intake of plant sourced nitrate was 61 [45–88] mg/day, vegetable sourced nitrate was 40 [28–57] mg/day, animal-sourced nitrate was 2.8 [1.8, 4.2] mg/day and processed meat-sourced nitrate was 0.68 [0.28, 1.29] mg/day (Table 1). Of total nitrate intake, plant-sourced nitrate contributed 86% (of this, vegetable-sourced nitrate contributed 65%, fruit nitrate 14%, whole grain nitrate 7%), animal-sourced nitrate 4%, and processed meat 1.2%. The remaining 9% was from alcohol and discretionary foods. The primary contributors to vegetable-sourced nitrate intake were lettuce (39%), zucchini (19%), cabbage (17%), pumpkin (10%), spinach (9%), and potato (8%). The main contributors to animal-sourced nitrate were yoghourt (26%), lamb (24%), beef (23%), and chicken (5%). Participants in the highest quartile of plant sourced nitrate were more likely to be older, be more physically active, have completed University or an equivalent degree, and consume higher amounts of fish, vegetables, and fruits (Table 1). Over 5-year of follow-up, there was minimal change in source dependent nitrate intake (Median change [IQR]; plant-sourced nitrate: 0.97 [−13, 16] mg/day; vegetable-sourced nitrate: 1.32 [−10, 13] mg/day; animal-sourced nitrate 0.09 [−0.85, 1.16] mg/day; and processed meat nitrate: 0 [−0.38, 0.32] mg/day).

**Table 1:** Baseline characteristics of study population.

	Total population	Plant nitrate intake quartiles			
	*n* = 9,149	Q1	Q2	Q3	Q4
		*n* = 2,288	*n* = 2,287	*n* = 2,287	*n* = 2,287
Plant-nitrate intake (mg/day)	61 [45, 81]	35 [27, 41]	53 [49, 56]	69 [65, 75]	98 [88, 116]
Vegetable-nitrate intake (mg/day)	40 [28, 57]	21 [14, 26]	35 [30, 40]	48 [41, 54]	72 [61, 86]
Animal-nitrate intake (mg/day)	2.8 [1.8, 4.2]	2.2 [1, 3]	2.6 [1, 3]	3 [2, 4]	3.6 [2, 5]
Processed-meat nitrate intake (mg/day)	0.68 [0.28, 1.29]	0.60 [0.25, 1.17]	0.69 [0.30, 1.24]	0.74 [0.31, 1.34]	0.71 [0.27, 1.41]
Total nitrate intake (mg/day)	73 [56, 95]	44 [36, 51]	64 [59, 70]	82 [76, 88]	113 [101, 131]
Sex (male) *n* (%)	4,277 (46.7)	1,046 (45)	1,021 (44)	1,089 (47)	1,121 (49)
Age (years)	49 [40, 59]	46 [37, 56]	48 [40, 58]	49 [41, 59]	51 [42, 62]
BMI	26 [23, 29]	25 [23, 29]	26 [23, 29]	26 [23, 29]	26 [23, 29]
Overweight (BMI 25 to <30)	3,657 (39)	852 (37)	949 (41)	927 (40)	929 (40)
Obese (BMI >30)	2,002 (21)	459 (20)	477 (20)	538 (23)	528 (23)
Physical activity					
Sedentary (0 min/week)	1,528 (16.7)	480 (20.9)	385 (16.8)	357 (15.6)	306 (13.3)
Insufficient (1–150 min/week)	2,789 (30.4)	742 (32.4)	734 (32.0)	690 (30.1)	623 (27.2)
Sufficient (>150 min/week)	4,832 (52.8)	1,066 (46.5)	1,168 (51.0)	1,240 (54.2)	1,358 (59.3)
Diabetes					
Known diabetes mellitus	349 (3.8)	65 (2.8)	97 (4.2)	88 (3.8)	99 (4.3)
Impaired fasting glucose	528 (5.7)	122 (5.3)	134 (5.8)	132 (5.7)	140(6.1)
Impaired glucose tolerance	1,076 (11.7)	259 (11.3)	274 (11.9)	245 (10.7)	298 (13.0)
New diabetes mellitus	347 (3.7)	94 (4.1)	87 (3.8)	88 (3.8)	78 (3.4)
Normal glucose levels	6,849 (74.8)	1,748 (76.4)	1,695 (74.1)	1,734 (75.8)	1,672 (73.1)
Cholesterol	5.6 [4.9, 6.3]	5.5 [4.9, 6.3]	5.6 [4.9, 6.3]	5.6 [4.9, 6.3]	5.5 [4.9, 6.2]
Education status					
Never, primary or some high school	3,562 (38.9)	912 (39.8)	896 (39.1)	872 (38.1)	882 (38.5)
Completed University or equivalent	5,587 (61.0)	1,376 (60.1)	1,391 (60.8)	1,415 (61.8)	1,405 (61.4)
Marital status					
Single	789 (8.6)	255 (11.1)	188 (8.2)	180 (7.8)	166 (7.2)
Married	6,644 (72.6)	1,555 (67.9)	1,680 (73.4)	1,730 (75.6)	1,679 (73.4)
*De facto*	458 (5.0)	107 (4.6)	118 (5.1)	108 (4.7)	125 (5.4)
Divorced	556 (6.0)	165 (7.2)	146 (6.3)	116 (5.0)	129 (5.6)
Separated	236 (2.58)	79 (3.4)	47 (2.0)	51 (2.2)	59 (2.5)
Widowed	466 (5.0)	127 (5.5)	108 (4.7)	102 (4.4)	129 (5.6)
Smoking status					
Never	5,029 (54.9)	1,265 (55.2)	1,233 (53.9)	1,265 (55.3)	1,266 (55.3)
Former	2,653 (29.0)	564 (24.6)	659 (28.8)	718 (31.3)	712 (31.1)
Current	1,467 (16.0)	459 (20.0)	395 (17.2)	304 (13.2)	309 (13.5)
SEIFA	1,031 [966, 1,075]	1,027 [971, 1,075]	1,035 [974, 1,080]	1,042 [971, 1,079]	1,020 [962, 1,074]
Income					
$1,500+ per week	1,643 (17.9)	404 (17.6)	422 (18.4)	447 (19.5)	370 (16.1)
$800–1,499 per week	2,696 (29.4)	651 (28.4)	714 (31.2)	693 (30.3)	638 (27.9)
$600–799 per week	1,265 (13.8)	328 (14.3)	324 (14.1)	305 (13.3)	308 (13.4)
$400–599 per week	1,386 (15.1)	343 (14.9)	325 (14.2)	325 (14.2)	393 (17.1)
$200–399 per week	1,477 (16.1)	376 (16.4)	338 (14.7)	360 (15.7)	403 (17.6)
$1–199 per week	682 (7.4)	186 (8.1)	164 (7.1)	157 (6.8)	175 (7.6)
Dietary characteristics					
Energy (kj/day)	7,973 [6,277, 10,056]	6,816 [5,348, 8,650]	7,595 [6,120, 9,418]	8,281 [6,734, 10,305]	9,309 [7,470, 11,588]
Total fish intake (g/day)	25 [13, 44]	17 [8, 30]	23 [12, 39]	29 [16, 46]	36 [20, 61]
Red meat intake (g/day)	70 [40, 113]	58 [32, 97]	67 [39, 106]	77 [46, 115]	83 [47, 132]
Processed meat intake (g/day)	18.2 [8, 32]	15 [7, 29]	18 [8, 32]	19 [9, 34]	18.8 [7, 35]
Dietary fibre intake (g/day)	21 [16, 27]	15 [11, 19]	19 [15, 23]	23 [19, 28]	28 [23, 35]
Saturated FA (g/day)	28 [20, 39]	26 [18, 36]	27 [19, 37]	28 [21, 39]	31 [22, 42]
Polyunsaturated FA (g/day)	11 [7, 15]	9 [6, 13]	10 [7, 15]	11 [8, 16]	13 [9, 17]
Monosaturated FA (g/day)	25 [18, 33]	22 [16, 30]	24 [18, 32]	26 [19, 34]	28 [21, 38]
Fruit intake (g/day)	264 [145, 403]	149 [92, 253]	238 [141, 341]	307 [194, 425]	396 [262, 551]
Vegetable intake (g/day)	154 [107, 207]	87 [57, 118]	135 [108, 167]	172 [141, 209]	233 [189, 285]
Alcohol intake (g/day)	5.7 [0.6, 18]	4.5 [0.5, 17]	6 [0.8, 19]	6.7 [0.6, 19]	5.7 [0.5, 18]

Median [IQR], *n* (%). BMI, Body mass index; FA, Fatty acids; IQR, Interquartile range; MET, Metabolic equivalent; g/day, grams per day; kJ/d, Kilojoules per day.

### 3.2 Association between nitrate intake and dementia-related mortality in the whole cohort

Over 17 years of follow-up, 93 (1.0%) dementia mortality cases were recorded out of 1,237 (13.5%) total deaths. Participants in quartile 4 of plant sourced nitrate intake (median intake of 98 mg/day) had a 58% lower risk of dementia-related mortality [Table 2; Model 2 HR (95% CI): 0.42 (0.21, 0.82)] and a 57% lower risk after further adjusting for dietary confounders [Table 2; Model 3b: 0.43 (0.22, 0.87)], compared to the participants in quartile 1 (median intake of plant sourced nitrate 35 mg/day). Similarly, for vegetable-sourced nitrate intake, participants in quartile 4 (median intake of 72 mg/day) had a 67% lower risk of dementia-related mortality [Table 2; Model 2: 0.33, (0.17, 0.64)] and a 66% lower risk after further adjusting for dietary confounders [Table 2; Model 3b: 0.34 (0.17, 0.66)], compared to those in quartile 1 (median intake 20 mg/day). In sensitivity analyses, the associations of plant- and vegetable-sourced nitrate with dementia-related mortality remained robust with model 3b that involved further adjustment for intakes of flavonoids, vitamin C, and fibre [HR_Q4vsQ1_ (CI 95%): 0.36 (0.16, 0.82) for plant-sourced nitrate, and 0.28 (0.14, 0.59) for vegetable-sourced nitrate]. There was no association observed for intake of animal-sourced nitrate and dementia-related mortality (Table 2). However, for processed meat-sourced nitrate intake, participants in quartile 4 (median intake of 1.93 mg/day) had double the risk of dementia-related mortality [Table 2; Model 3b HR (95% CI): 2.10 (1.07, 4.12)], compared to the participants in quartile 1 (median intake of processed meat-sourced nitrate 0.13 mg/day) after adjustment for dietary confounders. No effect modification was observed when analyses were stratified by sex. Participants in quartile 4 of vegetable-sourced nitrate had a lower risk of dementia-related mortality in both males [Model 2; HR_Q4vsQ1_ (95% CI): 0.38 (0.15, 0.97)] and females [0.32 (0.12, 0.84)].

**Table 2:** Hazard ratios of dementia related mortality by quartiles of source-dependent dietary nitrate intake.

Plant sourced nitrate intake quartiles	Q1	Q2	Q3	Q4
	*n* = 2,288	*n* = 2,287	*n* = 2,287	*n* = 2,287
Intake (mg/day)^*^	35 [27, 41]	53 [49, 56]	69 [65, 75]	98 [88, 116]
No of events	21	26	30	16
Model 1	Ref	1.04 (0.58, 1.85)	1.15 (0.66, 2.02)	0.44 (0.23, 0.86)
Model 2	Ref	1.07 (0.59, 1.93)	1.23 (0.69, 2.19)	0.42 (0.21, 0.82)
Model 3a	Ref	1.05 (0.58, 1.90)	1.16 (0.65, 2.10)	0.38 (0.18, 0.76)
Model 3b	Ref	1.10 (0.60, 2.01)	1.26 (0.70, 2.27)	0.43 (0.22, 0.87)
Vegetable sourced nitrate intake quartiles	Q1	Q2	Q3	Q4
	*n* = 2,288	*n* = 2,287	*n* = 2,287	*n* = 2,287
Intake (mg/day)^*^	20 [13, 24]	34 [31, 37]	47 [44, 51]	72 [63, 86]
No of events	26	22	30	15
Model 1	Ref	0.81 (0.45, 1.43)	0.85 (0.50, 1.45)	0.34 (0.18, 0.65)
Model 2	Ref	0.83 (0.46, 1.48)	0.88 (0.51, 1.51)	0.33 (0.17, 0.64)
Model 3a	Ref	0.82 (0.45, 1.46)	0.85 (0.49, 1.48)	0.31 (0.16, 0.61)
Model 3b	Ref	0.83 (0.46, 1.49)	0.88 (0.50, 1.53)	0.34 (0.17, 0.66)
Animal sourced nitrate intake quartiles	Q1	Q2	Q3	Q4
	*n* = 2,288	*n* = 2,287	*n* = 2,287	*n* = 2,287
Intake (mg/day)^*^	1.3 [0.9, 1.5]	2.3 [2.0, 2.5]	3.4 [3.1, 3.8]	5.4 [4.7, 6.4]
No of events	28	28	20	17
Model 1	Ref	1.24 (0.73, 2.11)	0.96 (0.54, 1.71)	0.76 (0.41, 1.39)
Model 2	Ref	1.21 (0.71, 2.06)	0.84 (0.46, 1.52)	0.74 (0.40, 1.37)
Model 3a	Ref	1.19 (0.69, 2.04)	0.80 (0.43, 1.50)	0.70 (0.36, 1.37)
Model 3b	Ref	1.23 (0.71, 2.14)	0.87 (0.46, 1.63)	0.81 (0.40, 1.61)
Processed meat sourced nitrate intake quartiles	Q1	Q2	Q3	Q4
	*n* = 2,289	*n* = 2,286	*n* = 2,287	*n* = 2,287
Intake (mg/day)^*^	0.13 [0.03, 0.20]	0.46 [0.36, 0.57]	0.94 [0.81, 1.09]	1.93 [1.54, 2.63]
No of events	27	25	16	25
Model 1	Ref	1.06 (0.61, 1.83)	0.84 (0.45, 1.59)	1.57 (0.89, 2.74)
Model 2	Ref	1.21 (0.69, 2.11)	0.86 (0.45, 1.64)	1.60 (0.90, 2.86)
Model 3a	Ref	1.24 (0.71, 2.19)	0.91 (0.47, 1.74)	1.84 (0.97, 3.50)
Model 3b	Ref	1.23 (0.69, 2.17)	0.94 (0.49, 1.81)	2.10 (1.07, 4.12)

Hazards Ratio (95% CI) for dementia related mortality for 17 years were obtained from Cox proportional hazards models with the exposure fitted as a quartile variable. The Hazards Ratio for exposure fitted as quartiles are reported for the median intake in each quartile(Q) relative to the median intake in Q1. Model 1 adjusted for age & sex; model 2 adjusted for all covariates in model 1 plus physical activity levels, level of education, body mass index, smoking status, marital status, alcohol intake, income, SEIFA, prevalent diabetes mellitus, and cholesterol; model 3a adjusted for all covariates in model 2 plus energy intake; when plant and vegetable sourced nitrate were the exposures of interest, model 3b adjusted for all covariates in model 2 plus intake (g/day) of red meat, fish, saturated fatty acids, polyunsaturated fatty acids, and monosaturated fatty acids and when naturally occurring animal and processed meat sourced were exposures of interest, model 3b adjusted for all covariates in model 2 plus saturated fatty acids, polyunsaturated fatty acids, monosaturated fatty, and intake of vegetables.

### 3.3 Association between nitrate intake and dementia-related mortality in participants with and without diabetes mellitus

Participants with DM (7.6%) and pre-diabetes (17.5%) were more likely to be older, were less likely to have completed a university or equivalent degree and were more likely to be male than participant without DM and pre-diabetes. (Supplementary Table 1). In participants with DM and pre-diabetes, a statistically significant 74 and 73% lower risk of dementia-related mortality was seen for participants with the highest, compared to the lowest, intakes of plant-sourced, and vegetable-sourced nitrate, respectively (Table 3; Model 2). For participants without DM, a statistically significant 67 and 72% lower risk of dementia-related mortality was seen for participants with the highest, compared to the lowest, intakes of vegetable-sourced, and naturally occurring animal-sourced nitrate, respectively (Table 3; Model 2 and Model 3a). On an absolute scale, participants with DM had a higher risk of dementia (Supplementary Table 2). The difference (quartile 4—quartile 1) in the 17-year predicted risk (adjusted for demographics and lifestyle risk factors) of dementia-related mortality was 1.43% for males with DM and 0.93% for males without DM, whilst for females with DM it was 2.31 and 0.47% for females without DM (Supplementary Table 2).

**Table 3:** Hazard ratios of dementia related mortality by quartiles of dietary nitrate intake stratified by prevalent diabetes mellitus and pre-diabetes.

Prevalent diabetes mellitus and pre-diabetes					No prevalent diabetes mellitus and pre-diabetes			
Plant sourced nitrate intake quartiles	Q1	Q2	Q3	Q4	Q1	Q2	Q3	Q4
	*n* = 575	*n* = 575	*n* = 575	*n* = 575	*n* = 1,713	*n* = 1,712	*n* = 1,712	*n* = 1,712
Intake (mg/day)^*^	36 [27, 42]	54 [50, 57]	70 [66, 76]	101 [90, 119]	35 [27, 40]	53 [49, 56]	69 [65, 74]	97 [87, 114]
No of events	12	13	16	5	10	12	14	11
Model 1	Ref	1.20 (0.54, 2.63)	1.27 (0.59, 2.69)	0.32 (0.11, 0.91)	Ref	0.89 (0.38, 2.07)	1.08 (0.48, 2.45)	0.56 (0.23, 1,13)
Model 2	Ref	1.17 (0.51, 2.67)	1.19 (0.54, 2.62)	0.26 (0.08, 0.77)	Ref	1.08 (0.45, 2.59)	1.26 (0.54, 2.96)	0.58 (0.24, 1.43)
Model 3a	Ref	1.23 (0.54, 2.81)	1.33 (0.59, 2.97)	0.31 (0.10, 0.96)	Ref	1.01 (0.42, 2.45)	1.12 (0.47, 2.68)	0.44 (0.17, 1.17)
Model 3b	Ref	1.22 (0.53, 2.82)	1.25 (0.56, 2.79)	0.27 (0.08, 0.84)	Ref	1.05 (0.43, 2.55)	1.30 (0.55, 3.11)	0.60 (0.23, 1.53)
Vegetable sourced nitrate intake quartiles	Q1	Q2	Q3	Q4	Q1	Q2	Q3	Q4
	*n* = 575	*n* = 575	*n* = 575	*n* = 575	*n* = 1,713	*n* = 1,712	*n* = 1,712	*n* = 1,712
Intake (mg/day)^*^	20 [14, 24]	35 [31, 38]	48 [45, 53]	75 [65, 91]	20 [13, 24]	34 [31, 37]	47 [44, 51]	71 [62, 85]
No of events	15	13	12	6	12	10	17	8
Model 1	Ref	0.94 (0.44, 1.97)	0.72 (0.33, 1.55)	0.32 (0.12, 0.83)	Ref	0.83 (0.35, 1.93)	0.99 (0.47, 2.10)	0.36 (0.14, 0.88)
Model 2	Ref	0.99 (0.46, 2.14)	0.69 (0.31, 1.52)	0.27 (0.10, 0.74)	Ref	0.78 (0.33, 1.86)	0.98 (0.45, 2.13)	0.33 (0.13, 0.85)
Model 3a	Ref	1.03 (0.48, 2.22)	0.77 (0.34, 1.72)	0.31 (0.11, 0.85)	Ref	0.74 (0.31, 1.75)	0.92 (0.42, 2.01)	0.26 (0.10, 0.70)
Model 3b	Ref	1.00 (0.46, 2.15)	0.69 (0.30, 1.55)	0.26 (0.09, 0.73)	Ref	0.78 (0.32, 1.86)	0.98 (0.44, 2.16)	0.34 (0.13, 0.90)
Animal sourced nitrate intake quartiles	Q1	Q2	Q3	Q4	Q1	Q2	Q3	Q4
	*n* = 575	*n* = 575	*n* = 575	*n* = 575	*n* = 1,713	*n* = 1,712	*n* = 1,712	*n* = 1,712
Intake (mg/day)^*^	1.3 [0.9, 1.5]	2.2 [2.0, 2.5]	3.5 [3.1, 3.8]	5.3 [4.7, 6.3]	1.3 [0.9, 1.6]	2.3 [2.0, 2.5]	3.4 [3.1, 3.8]	5.4 [4.8, 6.5]
No of events	14	11	10	11	13	18	10	6
Model 1	Ref	1.02 (0.46, 2.27)	0.97 (0.43, 2.21)	1.07 (0.48, 2.36)	Ref	1.49 (0.72, 3.05)	0.95 (0.41, 2.19)	0.47 (0.18, 1.26)
Model 2	Ref	0.92 (0.40, 2.07)	0.76 (0.33, 1.78)	0.92 (0.41, 2.08)	Ref	1.49 (0.71, 3.13)	0.69 (0.29, 1.67)	0.41 (0.15, 1.11)
Model 3a	Ref	1.09 (0.47, 2.52)	1.10 (0.44, 2.75)	1.41 (0.56, 3.52)	Ref	1.39 (0.65, 2.92)	0.57 (0.23, 1.41)	0.28 (0.09, 0.83)
Model 3b	Ref	1.18 (0.51, 2.76)	1.20 (0.48, 3.00)	1.58 (0.63, 3.94)	Ref	1.38 (0.64, 2.94)	0.59 (0.23, 1.47)	0.31 (0.10, 0.95)
Processed meat sourced nitrate intake quartiles	Q1	Q2	Q3	Q1	Q1	Q2	Q3	Q1
	*n* = 575	*n* = 575	*n* = 575	*n* = 575	*n* = 1,721	*n* = 1,704	*n* = 1,712	*n* = 1,712
Intake (mg/day)^*^	0.1 (0.0, 0.1)	0.4 (0.3, 0.5)	0.9 (0.8, 1.0)	1.9 (1.5, 2.6)	0.1 (0.0, 0.2)	0.4 (0.3, 0.5)	0.9 (0.8, 1.0)	1.9 (1.5, 2.6)
No of events	13	15	8	10	14	10	8	15
Model 1	Ref	1.36 (0.64, 2.88)	1.05 (0.42, 2.58)	1.41 (0.61, 3.27)	Ref	0.71 (0.31, 1.62)	0.64 (0.26, 1.57)	1.47 (0.69, 3.14)
Model 2	Ref	1.52 (0.70, 3.28)	1.04 (0.41, 2.60)	1.28 (0.53, 3.09)	Ref	0.90 (0.37, 2.16)	0.62 (0.24, 1.57)	1.58 (0.68, 3.65)
Model 3a	Ref	1.73 (0.79, 3.77)	1.33 (0.51, 3.43)	2.08 (0.79, 5.47)	Ref	0.90 (0.37, 2.16)	0.61 (0.24, 1.57)	1.55 (0.62, 3.84)
Model 3b	Ref	1.77 (0.80, 3.89)	1.40 (0.54, 3.64)	2.55 (0.92, 7.06)	Ref	0.89 (0.37, 2.14)	0.62 (0.24, 1.60)	1.71 (0.66, 4.37)

Hazards Ratio (95% CI) for dementia related mortality for 17 years were obtained from Cox proportional hazards models with the exposure fitted as a quartile variable. The Hazards Ratio for exposure fitted as quartiles are reported for the median intake in each quartile (Q) relative to the median intake in Q1. Model 1 adjusted for age & sex; model 2 adjusted for all covariates in model 1 plus physical activity levels, level of education, body mass index, smoking status, marital status, alcohol intake, income, SEIFA, and cholesterol; model 3a adjusted for all covariates in model 2 plus energy intake; when plant and vegetable sourced nitrate were the exposures of interest, model 3b adjusted for all covariates in model 2 plus intake (g/day) of red meat, fish, saturated fatty acids, polyunsaturated fatty acids, and monosaturated fatty and when naturally occurring animal and processed meat sourced were exposures of interest, model 3b adjusted for all covariates in model 2 plus saturated fatty acids, polyunsaturated fatty acids, monosaturated fatty, and intake of vegetables.

## 4 Discussion

In this cohort study of 9,149 participants followed for up to 17 years, the habitual intake of plant- and vegetable-sourced nitrate was associated with a lower risk of dementia mortality, whilst processed meat-sourced nitrate intake was associated with a higher risk of dementia-related mortality. Furthermore, the inverse association between a habitual intake of vegetable-sourced nitrate and dementia-related mortality did not differ in participants with and without DM. Given that participants with DM are at a higher risk of dementia, our findings suggest that this may be an important group to target to increase their intake of nitrate-rich vegetables.

We observed that a higher plant- and vegetable-sourced nitrate intake was associated with a 58–67% lower risk of dementia mortality compared to participants with a low intake. To our knowledge, the association between different sources of nitrate intake and dementia-related mortality remained unexplored previously. However, a recent study in the population-based Rotterdam cohort consisting of 9,543 participants with a mean age of 64 years showed an association between vegetable-derived nitrate and lower risk of incident dementia [HR: 0.92 (0.86, 0.97)] over a mean follow-up period of 14.5 years (38). There is also mounting evidence that certain dietary patterns, namely the Mediterranean Diet (MedDiet), Combination of MedDiet-dietary approaches to stop hypertension (DASH) Intervention for Neurodegenerative Delay (MIND), and Japanese diets, are associated with a lower risk of dementia (39, 40). These dietary approaches have several protective components and are all high in plant-sourced dietary nitrate. A meta-analysis of studies investigating higher adherence to the Mediterranean Diet and risk of Alzheimer’s disease (AD), the most common dementia subtype, has reported an 11% lower risk of AD [Relative Risk (RR): 0.89 (0.84, 0.93)] compared to lower Mediterranean Diet adherence (41). A recent study of 60,298 participants from the United Kingdom Biobank observed a 23% lower risk of incident dementia [HR: 0.77 (0.65, 0.91)] in participants with higher MedDiet adherence after multivariable adjustment (42). The Australian Personality and Total Health (PATH) Through Life cohort with 12 years of follow-up reported a 53% lower risk of cognitive decline for participants in the highest tertile of MIND diet adherence compared to the lowest tertile [Odds Ratio (OR): 0.47 (0.24, 0.91)] (43). Furthermore, the Rush Memory and Ageing Project with an average follow-up period of 4.5 years observed that the participants in the highest tertile of MIND diet adherence had a 53% reduced risk of AD [HR: 0.47 (0.29, 0.76)], whilst participants in the middle tertile had a 35% lower risk of AD [HR: 0.65 (0.44, 0.98)]. Moreover, in the Ohsaki Cohort 2006 study with a follow-up period of 13 years, the authors reported that participants in the highest tertile of Japanese diet, a diet which comprises higher intake of seaweed, vegetables, and fish, had a 21% lower risk of incident dementia compared to the lowest tertile [HR: 0.79 (0.66, 0.95)] (40). Common components of MedDiet, MIND, and Japanese diet are green leafy vegetables and seaweed which are high in dietary nitrate.

The primary sources of dietary nitrate are plant-based foods (mainly vegetables), water, and meat. These sources differ in their nitrate content considerably, which is regulated in most countries. The Scientific Committee for Food (SCF), in 1997, and the Joint Food and Agriculture Organization/World Health Organization (WHO) Expert Committee on Food Additives (JECFA), in 2003, set the Acceptable Daily Intake (ADI) of nitrate as 0–3.7 mg/kg body weight (~260 mg/70 kg adult) on the basis of a chronic feeding study in rats with unpublished data (27). The European Food Safety Authority carried out a risk assessment of nitrate intake in 2008, which was reviewed and accepted in 2015 (44). Importantly, the ADI guidelines do not distinguish between sources of nitrate intake. The ADI of nitrate can be surpassed by intake of a single serve of nitrate-rich vegetables; for example, a single serve of rocket (80 g) comprises ~360 mg nitrate (28). Notably, clinical trials have observed that the ADI of nitrate of ~260 mg/day for a 70 kg adult was associated with beneficial effects on vascular function and blood pressure (45). Also, individuals following the DASH diet, which is rich in vegetables might consume ~1,000 mg/day of nitrate (46). Furthermore, a systematic review of 55 observational studies which assessed daily nitrate intake in adults, reported a median intake in healthy participants of 108 [87–145 mg/day] and for patient population of 110 [89–153] mg/day from studies that included individuals who developed diseases during follow-up (47). In Japan, high nitrate intake diets contain approximately 1,100 mg of nitrate/adult/day (48). However, the median nitrate intake in this Australian cohort (61 mg/day) is considerably less compared to both the ADI and the nitrate dose of ~260 mg/day demonstrated to have beneficial effects on the vascular function in clinical trials. This intake difference could explain why we only observed a lower risk of dementia related mortality in the highest quartile (98 mg/day). We observed a lower risk of dementia in the highest nitrate intake quartile, but such an association was not observed in the moderate nitrate intake quartile. Nevertheless, the median nitrate intake in this cohort was relatively low compared to other studies that investigated the association of dietary nitrate and cardiovascular disease: 61 mg/day vs. 79–128 mg/day (49–52), this is approximately one cup of raw green leafy vegetables or half a cup of cooked green leafy vegetables per day. Future studies are required to ascertain the optimal dosage of dietary nitrate to reduce the risk of dementia in an ageing population.

The mechanism via which dietary nitrate may positively impact the risk of dementia is hypothesised to be via effects on NO. Dietary nitrate improves endogenous NO levels via the nitrate-nitrite-NO-pathway, which is associated with beneficial effects on vascular health (53, 54). After ingestion of dietary nitrate, ~75% of nitrate is excreted through kidneys, whilst ~25% is taken up by salivary glands and converted to nitrite by the anaerobic bacteria present in the clefts of the tongue surface. The enteric bacterial nitrite reductase, along with low pH in the stomach, reduces nitrite to NO. The remaining nitrate and nitrite are recycled through the enterosalivary nitrate-nitrite-NO-pathway. Dietary nitrate has been found to reduce cardiovascular risk factors such as blood pressure, endothelial dysfunction, arterial stiffness, and platelet aggregation, by increasing NO through the nitrate-nitrite-NO pathway. A meta-analysis has supported the association between nitrate intake and cardiovascular health (55). Mid-life vascular risk factors have also been linked to late-life brain health, and brain vascular dysregulation has been suggested to be an early sign of AD (56, 57). Studies have also shown that endothelial-derived NO may prevent tau phosphorylation, which is a hallmark of AD (58).

We observed that a higher processed meat-sourced nitrate intake was associated with double the risk of dementia mortality compared to participants with a low intake. To our knowledge this is the first study to investigate nitrate intake from processed meat and dementia mortality. However, processed meat intake was observed to be associated with a higher risk of all-cause dementia cases in the United Kingdom Biobank cohort (59). The preservation of processed meat products with nitrate and nitrite salts is speculated to contribute to the negative health outcomes of processed meat consumption. Nitrate, through conversion to nitrite, can react with amines or amides to form genotoxic, neurodegenerative, and carcinogenic *N*-nitroso compounds. However, it should be noted that whether the observed association in this study is due to the presence of nitrate as an allowed additive in processed meat is unclear as processed meat contains other potential harmful compounds such as polycyclic aromatic hydrocarbons and heterocyclic aromatic amines (60), which could not be accounted for in the analyses. Notably, there was no evidence for detrimental effects of naturally occurring animal-sourced nitrate and dementia-related mortality in this study. Indeed, higher animal-sourced nitrate was associated with reduced risk amongst those without DM. However, we acknowledge that intakes of animal sourced nitrate were low compared to plant sourced nitrate in the cohort and so these findings must be interpreted with caution.

The link between DM and dementia is now well-established (61). Insulin resistance, a typical feature of type 2 DM, causes impaired glucose metabolism in the brain leading to chronic neuroinflammation (62). Furthermore, insulin resistance can contribute to formation of amyloid plaques and neurofibrillary tangles, pathological hallmarks of AD (63). The association between DM and dementia could also be due to the two-fold higher risk of a wide range of cardiovascular diseases in individuals with DM (64). As robust evidence from clinical trials demonstrates that intake of dietary nitrate is beneficial to cardiovascular health (45), data were stratified by presence of DM to explore if dietary nitrate confers protection against dementia in this high-risk population. The 17-year predicted risk of dementia-related mortality (adjusted for lifestyle-risk factors) for people with DM and pre-diabetes in the lowest vegetable-sourced nitrate intake quartile was ~3.42 (for females) or ~ 2.09 (for males) was higher than their counterparts in the highest vegetable-sourced nitrate intake quartile ~1.11 (for females) or ~ 0.66 (for males). Thus, we might expect to prevent more cases of dementia if people with DM and pre-diabetes increased their intake of nitrate-rich vegetables.

The current study has some limitations that require consideration when interpreting the findings. Primarily, we only identified dementia cases from administrative data from a single source, and are therefore likely to have missed incident dementia cases, potentially introducing selection and misclassification bias (65). Furthermore, given the observational study design, we cannot infer causality. We also cannot disregard any unmeasured confounding factors. There is also a possibility of recall bias since this is questionnaire-based dietary data and estimating nitrate intake from database may not include uncommon high nitrate foods, also, does not account for factors that determine nitrate levels of vegetables such as soil type, growing conditions, intensity of sunlight, and storage conditions. We do not attribute observed benefits entirely to the nitrate intake because the correlation between intake of vegetable-sourced nitrate and overall consumption of vegetables was strong (ρ = 0.79), which contain other beneficial compounds that can mitigate the risk of dementia-related mortality. Moreover, we only considered dietary intake data captured at baseline in our analyses. Any changes to dietary habits over time, would likely have attenuated the observed associations. Also, nitrate from water was not included as we did not know the nitrate levels of water the participants consumed. Additionally, other than being excluded at baseline for a dementia diagnosis, there was no information on cognitive impairment at baseline, which may have impacted ability to recall dietary habits accurately. Also, we could not adjust for carriage of the ε4 allele of the apolipoprotein E gene (*APOE*), the most common genetic risk factor for AD (66), and we could not distinguish between dementia subtypes. The current study is also limited by using dementia-related mortality as a proxy for dementia diagnosis. This meant only dementia in deceased participants was identified. Moreover, not all cases of dementia are identified on death certificates and there has been some increased identification on certificates over time (67).

Nevertheless, this cohort study has several strengths. The follow-up period of 17 years was of importance due to the prolonged nature of the dementia. The mean enrolment age (~49 years) together with the length of follow up has allowed for the examination of dietary nitrate intake from different sources in association with mid-life dementia risk factors. Consideration of mid-life risk factors along with exposure is of utmost importance in assessing late-life dementia risk as pathological changes begin to appear 10–20 years before the onset of clinical symptoms of dementia (68). Importantly, the association between higher nitrate intake and dementia-related mortality remained significant even with adjustment for dietary confounders and lifestyle factors. Finally, we used the latest comprehensive nitrate databases to calculate dietary nitrate intake from different sources (13, 28).

## 5 Conclusion

In this large cohort study, we observed that a higher habitual intake of plant-sourced nitrate, specifically from vegetables, was significantly associated with a lower risk of dementia-related mortality, whilst higher intakes of processed meat-sourced nitrate were associated with a higher risk of dementia. These findings suggest that encouraging the intake of nitrate-rich vegetables, may lower the risk of dementia-related mortality for those with and without (pre-) diabetes mellitus.

## Data availability statement

The data analysed in this study are subject to the following licences/restrictions: the data presented in this article are not readily available since it belongs to the Baker Heart and Diabetes Institute. There are restrictions for the availability of these data, which were used under licence for the current study. Raw data are not publicly available. However, data described in the manuscript, codebook, and analytic code will be made available upon request and approval of the AusDiab Steering Committee. Requests to access these datasets should be directed to jonathan.shaw@baker.edu.au.

## Ethics statement

The studies involving humans were approved by International Diabetes Institute Ethics Committee. The studies were conducted in accordance with the local legislation and institutional requirements. The participants provided their written informed consent to participate in this study.

## Author contributions

AR: Conceptualization, Formal analysis, Methodology, Writing – original draft. NB: Conceptualization, Formal analysis, Methodology, Supervision, Writing – review & editing. LZ: Writing – review & editing, Methodology. SR-B: Writing – review & editing. KM: Formal analysis, Writing – review & editing. SR-S: Supervision, Writing – review & editing. SG: Supervision, Writing – review & editing. LB: Writing – review & editing. DM: Writing – review & editing. JS: Writing – review & editing. RD: Writing – review & editing. KA: Writing – review & editing. JL: Writing – review & editing. JH: Supervision, Writing – review & editing. CB: Conceptualization, Formal analysis, Methodology, Supervision, Writing – original draft, Writing – review & editing.
