# Supplementary material for: Plant but not animal sourced nitrate intake is associated with lower dementia-related mortality in the Australian Diabetes, Obesity, and Lifestyle Study
Source: Front Nutr. 2024 Aug 21;11:1327042. doi: 10.3389/fnut.2024.1327042 (PMC11371772; doi:10.3389/fnut.2024.1327042)
Supplement: Supplementary file 1 [file Table_1.docx]

| **Supplementary Table 1: Baseline characteristics stratified by prevalent diabetes mellitus and pre-diabetes** | | |
| --- | --- | --- |
|  | **Prevalent diabetes & pre-diabetes n = 2,300** | **No prevalent diabetes & pre-diabetes n = 6,849** |
| **Sex (male) n (%)** | 1240 (53.9) | 3037 (44.3) |
| **Age (years)** | 57 [47, 67] | 46 [38, 56] |
| **BMI** | 28 [25, 32] | 25 [23, 28] |
| Overweight (BMI 25 to <30) | 945 (41.0) | 2712 (39.6) |
| Obese (BMI >30) | 865 (37.6) | 1137 (16.6) |
| **Physical activity** |  |  |
| **Sedentary (0 physical activity time)** | 459 (19.9) | 1069 (15.61) |
| **Insufficient (<150 min/week)** | 750 (32.6) | 2039 (29.77) |
| **Sufficient (>150 min/week)** | 1091 (47.4) | 3741 (54.6) |
| **Education status** |  |  |
| Never, Primary or some high school | 1042 (45.3) | 2520 (36.7) |
| Completed Uni or equivalent | 1258 (54.7) | 4329 (63.2) |
| **Marital status** |  |  |
| Single | 138 (6.0) | 651 (9.5) |
| Married | 1666 (72.4) | 4978 (72.6) |
| De facto | 68 (2.9) | 390 (5.6) |
| Divorced | 156 (6.7) | 400 (5.8) |
| Separated | 54 (2.3) | 182 (2.6) |
| Widowed | 218 (9.4) | 248 (3.6) |
| **Smoking status** |  |  |
| Never | 1150 (50.0) | 3879 (56.6) |
| Former | 827 (35.9) | 1826 (26.6) |
| Current | 323 (14.0) | 1144 (16.7) |
| **Cholesterol** | 5.7 [5.1, 6.4] | 5.5 [4.8, 6.2] |
| **SEIFA** | 1008 [962, 1067] | 1035 [971, 1079] |
| **Income** |  |  |
| $1500+ per week | 289 (12.5) | 1354 (19.7) |
| $800-1499 per week | 550 (23.9) | 2146 (31.3) |
| $600-799 per week | 290 (12.6) | 975 (14.2) |
| $400-599 per week | 398 (17.3) | 988 (14.4) |
| $200-399 per week | 528 (22.9) | 949 (13.8) |
| $1-199 per week | 245 (10.6) | 437 (6.3) |
| **Dietary characteristics** |  |  |
| Energy (kj/d) | 7786 [6141, 9716] | 8027 [6326, 10180] |
| Plant nitrate intake | 61 [46, 82] | 60 [44, 80] |
| Vegetable nitrate intake | 41 [28, 58] | 40 [28, 56] |
| Animal nitrate intake | 2.8 [1.8, 4.2] | 2.8 [1.8, 4.2] |
| Processed meat nitrate intake | 0.6 [0.2, 1.2] | 0.6 [0.2, 1.2] |
| Total nitrate intake | 73 [56, 97] | 73 [55, 94] |
| Total fish intake (g/day) | 26.1 [13, 46] | 25 [13, 43] |
| Red meat intake (g/day) | 72 [42, 118] | 70 [39, 111] |
| Processed meat intake (g/day) | 17 [7, 32] | 18 [8, 33] |
| Dietary fibre intake (g/day) | 21 [16, 27] | 21 [16, 27] |
| Saturated FA (g/day) | 27 [19, 36] | 29 [20, 39] |
| Polyunsaturated FA (g/day) | 11 [8, 15] | 11 [7, 15] |
| Monosaturated FA (g/day) | 24 [18, 32] | 25 [18, 34] |
| Fruit intake (g/day) | 267 [147, 409] | 263 [143, 402] |
| Vegetable intake (g/day)  Median [IQR], n (%). Abbreviations: BMI, body mass index; FA, fatty acids; IQR, interquartile range; g/day, grams per day; kj/day, kilojoules per day; n, number; mg/day, milligrams per day; %, percentage. | 148 [104, 202] | 144 [100, 196] |

**Supplementary Table 2. 17-year predicted risk of dementia related mortality**

| **Vegetable sourced nitrate intake** | | | |
| --- | --- | --- | --- |
|  | **Q1 risk (95% CI)** | **Q4 risk (95% CI)** | **Risk difference (%)** |
| **Male** |  |  |  |
| With diabetes | 2.09 (0.77, 3.42) | 0.66 (0.06, 1.26) | 1.43 |
| Without diabetes | 1.55 (0.72, 2.41) | 0.62 (0.17, 1.07) | 0.93 |
| **Female** |  |  |  |
| With diabetes | 3.42 (1.49, 5.36) | 1.11 (0.16, 2.08) | 2.31 |
| Without diabetes  The 17-year predicted risks (%) of dementia related mortality calculated from multivariable adjusted logistic regression models. | 0.75 (0.20, 1.30) | 0.28 (0.04, 0.51) | 0.47 |
